# Novel mTORC2/HSPB4 Interaction: Role and Regulation of HSPB4 T148 Phosphorylation

**DOI:** 10.3390/cells13232000

**Published:** 2024-12-04

**Authors:** Zachary B. Sluzala, Yang Shan, Lynda Elghazi, Emilio L. Cárdenas, Angelina Hamati, Amanda L. Garner, Patrice E. Fort

**Affiliations:** 1 Department of Ophthalmology & Visual Sciences, The University of Michigan, Ann Arbor, MI 48109, USA; zsluzala@umich.edu (Z.B.S.); shany@med.umich.edu (Y.S.); lelghazi@med.umich.edu (L.E.); hamatia@umich.edu (A.H.); 2 Interdepartmental Program in Medicinal Chemistry, The University of Michigan, Ann Arbor, MI 48109, USA; ecardena@med.umich.edu (E.L.C.); algarner@med.umich.edu (A.L.G.); 3 Department of Molecular & Integrative Physiology, The University of Michigan, Ann Arbor, MI 48109, USA

**Keywords:** sHSP, HSPB4, αA-crystallin, kinase, phosphorylation, neuroprotection, chaperone

## Abstract

HSPB4 and HSPB5 (α-crystallins) have shown increasing promise as neuroprotective agents, demonstrating several anti-apoptotic and protective roles in disorders such as multiple sclerosis and diabetic retinopathy. HSPs are highly regulated by post-translational modification, including deamidation, glycosylation, and phosphorylation. Among them, T148 phosphorylation has been shown to regulate the structural and functional characteristics of HSPB4 and underlie, in part, its neuroprotective capacity. We recently demonstrated that this phosphorylation is reduced in retinal tissues from patients with diabetic retinopathy, raising the question of its regulation during diseases. The kinase(s) responsible for regulating this phosphorylation, however, have yet to be identified. To this end, we employed a multi-tier strategy utilizing in vitro kinome profiling, bioinformatics, and chemoproteomics to predict and discover the kinases capable of phosphorylating T148. Several kinases were identified as being capable of specifically phosphorylating T148 in vitro, and further analysis highlighted mTORC2 as a particularly strong candidate. Altogether, our data demonstrate that the HSPB4-mTORC2 interaction is multi-faceted. Our data support the role of mTORC2 as a specific kinase phosphorylating HSPB4 at T148, but also provide evidence that the HSPB4 chaperone function further strengthens the interaction. This study addresses a critical gap in our understanding of the regulatory underpinnings of T148 phosphorylation-mediated neuroprotection.

## 1. Introduction

HSPB4 (αA-crystallin) is one of ten mammalian small heat shock proteins (sHSPs; HSPB1-10) [1,2]. sHSPs are characterized by a conserved “α-crystallin domain” flanked by less-well-conserved N- and C-terminal domains [3]. They also generally share a propensity to form large and polydisperse homo- and hetero-oligomers with other sHSPs [3,4]. sHSPs serve as chaperone proteins, preventing the misfolding and aggregation of their substrate proteins [4]. HSPB4, along with HSPB5 (αB-crystallin), together form the hetero-oligomeric “α-crystallin” and have historically been studied due to their chaperone function in the eye lens and their role in cataract prevention [5,6,7]. In part through this capacity to chaperone other proteins, sHSPs also possess anti-apoptotic and otherwise protective capabilities in several contexts, recently garnering increased attention for their roles in retinal neurodegenerative disorders [8].

HSPB4 and HSPB5 have both been shown to be expressed in multiple cell types of the retina, including in retinal pigment epithelial (RPE) cells [9,10,11,12,13,14,15,16], photoreceptor cytoplasm, inner and outer segments [15,17,18,19], astrocytes [20,21], Müller glial cells (MGC) [20,22,23], and retinal ganglion cells (RGC) [14,15,16,19,23,24]. They have been implicated in both neuronal and glial protection. In neurons, HSPB4 has been shown to protect photoreceptors in sympathetic ophthalmia [19] and during retinal detachment [25]. Both HSPB4 and HSPB5 are protective of RGCs after optic nerve axotomy [24] and after optic nerve crush in rats [26]. RGC death in rat glaucoma models is also reduced upon the intravitreal injection of HSPB5 [27]. shRNA knockdown of HSPB4, HSPB5, or both induces basal and stress-associated apoptosis in R28 retinal neuronal cells, while overexpression promotes survival under stress conditions [28]. In the glia, whole-body HSPB5-KO results in greater levels of cleaved caspase-3 and increased TUNEL staining in astrocytes, which is associated with worse outcomes in experimental autoimmune encephalomyelitis (EAE) [21]. Both HSPB4 and HSPB5 have also been shown to protect astrocytes from C2-ceramide and staurosporine-induced cell death [29,30], while HSPB4 has been shown to modulate inflammatory marker expression in both rMC-1 and primary MGCs [31]. These glial and neuronal mechanisms seem to be highly interconnected, as we recently demonstrated that RGCs can be protected by HSPB4 secreted by MGCs [23,32].

Phosphorylation regulates each of the HSPB4 and HSPB5 characteristic properties in both the retinal and non-retinal contexts, including their oligomerization [32,33,34,35], chaperone function [32,33,34,35,36,37,38], and protective capacity [23,29,30,32,39,40,41,42]. S122 phosphorylation of HSPB4 and S19, S45, and S59 phosphorylation of HSPB5 have been the primary targets of investigation, with S122 originally described as the only bona fide phosphorylation site in HSPB4. More recently, S/T148 (serine in rodents, threonine in humans) has been identified as another important phosphosite for HSPB4. S148 phosphorylation was first identified in mouse lens tissue using 2D gel electrophoresis [43]. Subsequently, S/T148 phosphorylation was reported in the retina using tandem mass spectrometry and found to be decreased in both animal models and human patients with diabetes, more substantially so in patients with diabetic retinopathy [23]. We recently characterized the biochemical, structural, and functional impacts of T148 phosphorylation, demonstrating that a phosphomimetic (T148D), but not a non-phosphorylatable (T148A) HSPB4 exhibited improved chaperoning of ADH, reduced stress-induced insolubility, and reduced oligomeric size [32]. We also demonstrated that WT or T148D HSPB4, but not T148A, could protect against retinal neuronal cell death, prevent mitochondrial Bax translocation, mitigate ER stress, and regulate inflammatory molecule expression [23,31,32]. Considering these numerous structural, functional, and neuroprotective implications of T148 phosphorylation, we sought to investigate which kinases are responsible for this post-translational modification. To this end, we utilized a combination of complementary approaches to identify T148-phosphorylating kinases and investigate the regulation of HSPB4 in neurons and glia.

## 2. Materials and Methods

### 2.1. Cell Culture

R28 (passage 55–70; Applied Biological Materials Inc., Richmond, BC, Canada) and MIO-M1 (passage 55–65; XPI, London, UK) cells were maintained in 5 mM glucose Dulbecco’s Modified Eagle Medium (DMEM; #10567-014; Gibco, Bleiswijk, The Netherlands) supplemented with 10% fetal bovine serum (FBS; #SH30071.03; Hyclone, South Logan, UT, USA) except where otherwise stated. Prior to their use in experiments, the R28 cells were neuronally differentiated as previously described [44] via plating on laminin-coated plates and supplementation with 25 mM 8-(4-chlorophenylthio) cAMP (8-CPT-cAMP; #C3912, MilliporeSigma, Darmstadt, Germany).

### 2.2. Electroporation-Based Transient Transfection

Cells were transfected using a Neon Transfection System (#MPK5000S; Invitrogen, Eugene, OR, USA) with plasmids coding for the expression of 3X-FLAG-tagged wild-type (WT), T148C, T148D, T148A, R21A, or R49A HSPB4 (5 μg DNA; pcDNA3.1(+) vector backbone [#V79020; Invitrogen, Eugene, OR, USA]), or an empty vector (EV) control. Transfection settings for the R28 cells were: 1600 V, 20 ms, 1 pulse. Transfection settings for the MIO-M1 cells were: 1400 V, 20 ms, 2 pulses. For the experiments, subsets of cells were subjected to “diabetes-like” stress (0.5–2 h incubation with 25 mM glucose DMEM + 10% FBS + 100 ng/mL TNF-α [#210-TA; R&D Systems, Minneapolis, MN, USA] following 24 h high-glucose conditioning; HG+TNF-α). This stress was chosen in part because TNF-α is one of the main cytokines found in excess in the retina and vitreous of human patients with diabetes, and in part because TNF-α is known to phosphorylate other sHSPs [45,46]. The short duration was chosen to avoid the downregulation of T148 phosphorylation seen in late-stage diabetes and DR (i.e., with extended exposure to these conditions).

### 2.3. Immunoprecipitation (IP) Experiments

For the PhAXA experiments, cells were subjected to 2 h of “diabetes-like” stress, harvested by scraping in a detergent-free lysis buffer (50 mM Tris pH 7.5, 150 mM NaCl, 10 mM MgCl_2_, 1× protease inhibitor cocktail [#11836170001; Roche, Mannheim, Germany], DI H_2_O) and lysed by passing through a 28.5 ga syringe. After clearing the lysate using centrifugation, an ATP methacrylate crosslinker [47] was added to a final concentration of 0.25 mM. Crosslinker-containing cell lysates were shaken at 30 °C for 1 h at 125 RPM. While the tubes shook, FLAG M2 Magnetic Resin (#M8823; MilliporeSigma, Darmstadt, Germany) was added to fresh 1.5-mL tubes and washed with 1× TBS. TBS was subsequently aspirated, and lysates were added to these tubes following shaking. The tubes were rotated end-over-end overnight at 4 °C for 3X-FLAG immunoprecipitation (IP). Full PhAXA outputs can be found in Supplemental File S1. For the other co-IP experiments, cells were exposed to 2 h of “diabetes-like” stress, harvested by scraping in an IP buffer (50 mM HEPES, 137.5 mM NaCl, 1 mM MgCl_2_, 1 mM CaCl_2_, 2 mM NaVO4, 10 mM NaPyroPhos, 10 mM NaF, 2 mM EDTA, 2 mM PMSF, 10 mM benzamidine, 10% glycerol, 1% NP-40, protease and phosphatase inhibitor tablets) and lysed using a syringe. Protein A Sepharose 6MB (#17-0469-01; GE Healthcare, Uppsala, Sweden) was used, bound with an antibody against Rictor (#04-1471; MilliporeSigma, Darmstadt, Germany), Raptor (#05-1470; MilliporeSigma, Darmstadt, Germany), or FLAG (DYKDDDDK Tag; CST, #2368). The Rictor IPs were repeated in the presence of the cell-permeable crosslinker Dithiobis (succinimidyl propionate) (DSP; #A35393; Thermo Fisher Scientific, San Jose, CA, USA). For the DSP crosslinker experiments, cells were incubated with 0.25 mM (DSP) (#A35393; Thermo Fisher Scientific, San Jose, CA, USA) concurrent with 30 min of “diabetes-like” stress at 37 °C, and subsequently for 15 min with 20 mM Tris in 1× PBS to halt further crosslinking. rProtein A/G Agarose (Marvelgent; #11-0210-005) was used for these co-IPs.

### 2.4. Sample Preparation and Immunoblot Analyses

Lysate protein concentrations were obtained using a detergent compatible (DC) protein assay (Bio-Rad, Hercules, CA, USA) with bovine serum albumin (BSA) standards (#23209; Thermo Fisher Scientific, San Jose, CA, USA). In total, 1000–2500 μg of protein were used for PhAXA and the other IP experiments, and 10–20 μg of protein were used for the input samples. Samples were prepared with an LDS sample buffer (#B0007; Novex, Carlsbad, CA, USA) supplemented with either BME- or DTT-reducing agents and subjected to an immunoblot analysis. Samples were run on 4–12% bis-tris plus gels (#NW04120; Invitrogen, Eugene, OR, USA) and transferred onto nitrocellulose membranes. Membranes were blocked using the EveryBlot blocking buffer (#12010020; Bio-Rad, Hercules, CA, USA) or 5% milk and imaged using a FluorChem E (Protein Simple) or Azure (c500) imaging system. Membranes were probed with antibodies against actin (1:5000; #3700; Cell Signaling Technologies [CST], Danvers, MA, USA), HSPB4 (1:1000; #ADI-SPA-221-F; Enzo, Farmingdale, NY, USA), FLAG (1:1000; “DYKDDDDK Tag” #14793S; CST, Danvers, MA, USA), Rictor (1:1000; #2140S; CST, Danvers, MA, USA), Raptor (1:1000; #2280S; CST, Danvers, MA, USA), mTOR (1:1000; #4517S; CST, Danvers, MA, USA), p-S2481 mTOR (1:1000; #2974S; CST, Danvers, MA, USA), and/or SIN1 (1:1000; #12860S; CST, Danvers, MA, USA). Fluorescent secondary antibodies from Jackson ImmunoResearch [JIR] were used (1:5000; #715-655-150 or #715-625-152; JIR, West Grove, PA, USA). For the PhAXA experiments, separate gels were stained with a Sypro Ruby gel stain (#S12000; Invitrogen, Eugene, OR, USA) for the collection of gel slices for liquid chromatography using a tandem mass spectrometry (LC-MS/MS) analysis. Gels were fixed at room temperature with 50% methanol, 7% acetic acid in ultrapure DI water for 30 min while shaking. The fix solution was replaced and incubated for an additional 30 min at room temperature while shaking. Gels were then incubated overnight at room temperature with the Sypro Ruby gel stain. The following day, the Sypro Ruby gel stain was removed, and gels were washed at room temperature 10% methanol, 7% acetic acid in ultrapure DI water for 30 min. Gels were then washed with ultrapure DI water twice for 5 min each before being imaged on a transilluminator (Ultra-Violet Products, Jena, Germany) using ultra-violet light for the gel slice collection. Slices were immediately stored at −80 °C until being submitted for the LC-MS/MS analysis by the Proteomics Resource Facility, a part of Biomedical Research Core Facilities at the University of Michigan.

### 2.5. In-Gel Digestion, Mass Spectrometry, & Database Search

Gel slices were destained with 30% methanol for 4 h. Upon reduction (10 mM DTT) and alkylation (65 mM 2-Chloroacetamide) of the cysteines, the proteins were digested overnight with 500 ng of sequencing grade, modified trypsin (Promega, Madison, WI, USA) at 37 °C. Peptides were extracted by incubating the gel with 150 µL of 60% acetonitrile/0.1% TFA for 30 min at room temperature. A second extraction with 150 µL of 100% acetonitrile/0.1% TFA was also performed. Both extracts were combined and dried in a vacufuge (Eppendorf, Enfield, CT, USA). The resulting peptides were dissolved in 9 µL of 0.1% formic acid/2% acetonitrile solution and 2 µL of the resulting peptide solution were resolved on a nano-capillary reverse phase column (Acclaim PepMap C18, 2 micron, 50 cm; Thermo Fisher Scientific, San Jose, CA, USA) using a 0.1% formic acid/acetonitrile gradient at 300 nL/min over a period of 90 min (2–25% acetonitrile in 35 min; 25–50% acetonitrile in 20 min, followed by a 90% acetonitrile wash for 5 min, and a further 30 min re-equilibration with 2% acetonitrile). Eluent was directly introduced into a *Q Exactive HF* mass spectrometer (Thermo Fisher Scientific, San Jose, CA, USA) using an EasySpray source. MS1 scans were acquired at a 60K resolution (AGC target = 3 × 10^6^; max IT = 50 ms). Data-dependent collision-induced dissociation MS/MS spectra were acquired on the 20 most abundant ions following each MS1 scan (NCE ~28%; AGC target 1 × 10^5^; max IT 45 ms). Proteins were identified using Proteome Discoverer (v2.4; Thermo Fisher Scientific, San Jose, CA, USA). The search parameters included an MS1 mass tolerance of 10 ppm and a fragment tolerance of 0.2 Da; two missed cleavages were allowed; the carbamidomethylation of cysteine (+57.012 Da) was considered a fixed modification, the oxidation of methionine (+15.994 Da), and the deamidation of asparagine and glutamine (+0.984 Da) were considered as variable modifications. The false discovery rate (FDR) was determined using Percolator and proteins/peptides with an FDR of ≤1% were retained for further analysis.

### 2.6. Bioinformatic Approaches

Phosphosite and candidate kinase predictions were made using several databases and programs, including KinasePhos2.0 [48], KinasePhos3.0 [49], NetPhos3.1 [50], iPhoPred [51], PhosphoSVM [52], ScanProsite [53], Group-Based Prediction System (GPS) 6.0 [54], YinOYang1.2 [55], the Human Protein Reference Database (HPRD) [56], the HPRD PhosphoMotif Finder [57], PhosphoNet [58], Phospho.ELM [59], Uniprot [60], PhosphoSitePlus [61] & the Kinase Library [62], and Scansite4.0 [63]. The full output readouts from each program can be found in Supplemental File S2. Kinase consensus sequences were obtained from https://kinase-library.phosphosite.org/site (accessed on 15 October 2024) and can be found in Supplemental Figure S1.

### 2.7. In Vitro Kinase Screens

A panel of kinases (100 or 10 nM; Thermo Fisher Scientific, San Jose, CA, USA) was screened against an HSPB4 peptide containing amino acids 142–154 (Pacific Immunology, Ramona, CA, USA) or the full-length recombinant WT and T148A HSPB4. Each kinase was incubated with a substrate (10 µM ATP + 10 µM peptide or 5 µM protein), an anti-ADP Eu antibody (2 nM, #R-8979; Thermo Fisher Scientific, San Jose, CA, USA), and an ADP tracer (8 nM or 40 nM for peptide and protein screens, respectively; #R-8981; Thermo Fisher Scientific, San Jose, CA, USA) in a buffer (50 mM HEPES pH 7.5, 0.01% BRIJ-35, 10 mM MgCl_2_ and 1 mM EGTA) overnight, with readings taken between 15 min and overnight. Controls with no peptide/protein and controls with no ATP were run for all the kinases. An ATP/ADP standard curve was run in the presence of a peptide or WT protein and used to convert the emission ratios to the product formed (ADP in µM) (Figure S1). Reactions were mixed in a plate shaker before and between readings. Readings were taken using Tecan SPARK under optimal gain settings.

### 2.8. Statistics & Data Analysis

Immunoprecipitated kinases were identified in PhAXA datasets by searching the resulting datasets for “kinase”. Kinases (and mTOR complex proteins) identified by <2 peptides were excluded from the analyses. Peptide spectrum matches (PSMs) and protein abundance values were normalized by separately summing the total PSM or abundance values in the WT-FLAG + crosslinker and T148C-FLAG + crosslinker conditions and dividing these summed values. The PSM and abundance values were then divided by the resulting normalization factors and 1 PSM was added to each normalized PSM value to allow for the fold-change (FC) calculation. The PSM FC values were calculated by dividing the “normalized PSM + 1” value for T148C-FLAG by the “normalized PSM + 1” value for WT-FLAG, and the abundance FC values, where possible, were similarly calculated. mTORC1/2 components were manually identified and analyzed the same way. All the immunoblots are representative of at least three experimental replicates. For the kinase screens, standard curves were used to convert the emission ratios to the ADP formed (in µM). Data were analyzed using a four-parameter fit using GraphPad Prism v. 10.2.2 (Graphpad, Boston, MA, USA).

## 3. Results

### 3.1. Phosphosite-Accurate Kinase-Substrate Crosslinking Assay (PhAXA)

**Figure 1:**
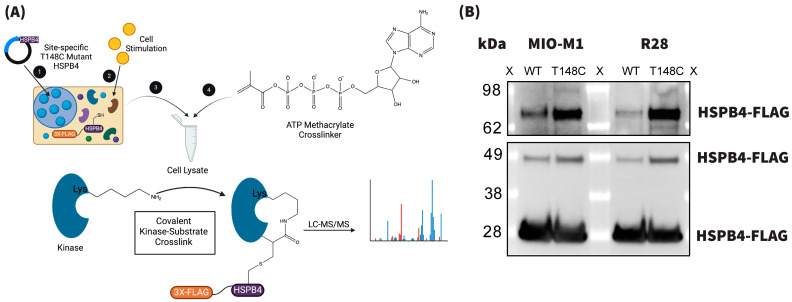
(**A**) Graphical representation of the PhAXA experimental process (adapted from [47]). (**B**) Representative PhAXA blot showing the band profiles in both the MIO-M1 and R28 cell line experiments. (X—marker; WT—wild-type).

We began our interrogation of T148 phosphorylation by utilizing a chemoproteomic method that we recently developed for identifying site-selective kinase–substrate interactions [47]. This technique, termed the phosphosite-accurate kinase–substrate crosslinking assay (PhAXA), utilizes an ATP–methacrylate crosslinker to allow for the covalent trapping of typically labile and transient kinase–substrate interactions (Figure 1A) [47,64]. Cross-linking occurs via the reaction between a cysteine residue, which is inserted at the phosphosite-of-interest, and a lysine residue within the ATP-binding pocket of a kinase, which is converted into an electrophilic moiety via binding to the ATP–methacrylate crosslinker. An advantage of this approach is its use of full-length proteins as bait, which are transiently overexpressed in cells to promote the formation of site-specific biomolecular interactions in the cell lysate context. We performed PhAXA in both R28 neuronal and MIO-M1 Müller glial cell lines to investigate the cell-type specific regulation of T148 and possible candidate kinase differences. A representative Western blot from the PhAXA experiments in each of these cell types is shown in Figure 1B. The presence [47,64] of higher molecular weight protein bands, specifically enriched in the T148C-FLAG+crosslinker condition, indicated successful crosslinking.

Following crosslinking and immunoprecipitation, the samples were run on gels and slices encompassing the entire gel lanes were collected for a subsequent LC-MS/MS analysis. Samples were submitted in this manner, as opposed to the submission of the protein-bound resin, to limit the identification of weak and non-crosslinker-mediated interactions. Due to the chaperone roles of HSPB4, we hypothesized that several non-kinase activity-associated protein–protein interactions would be identified, which the denaturation of the samples prior to MS/MS would limit. Indeed, even with this approach, we identified numerous non-kinases as being co-immunoprecipitated with HSPB4 (Supplemental File S1). The resulting datasets were therefore searched specifically for the identified kinases, which were the subject of further analyses. To limit false positives, we excluded from the analyses any kinase that was identified with only a single peptide and kinases that were not consistently identified in each experimental replicate. Of the remaining kinases, those that exhibited a >2 fold-change (FC) difference between the T148C and WT samples in peptide spectrum matches (PSMs) and/or protein abundance were considered as *bona fide* candidates for T148 phosphorylation.

**Figure 2:**
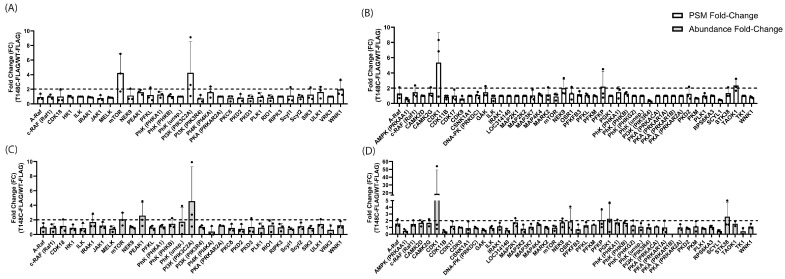
Kinases reproducibly identified via PhAXA in R28 (**A**,**C**) or MIO-M1 (**B**,**D**) cells. (**A**) R28 PSM fold-change; (**B**) MIO-M1 PSM fold-change; (**C**) R28 abundance fold-change; (**D**) MIO-M1 abundance fold-change. (n = 3 for each cell line) Error bars represent SD.

In total, 31 kinases were reproducibly observed in the R28 PhAXA experiments (n = 3). Of these, only two kinases (mTOR and PIK3C2A) met our criteria with respect to both PSM and abundance FC. mTOR displayed average PSM and abundance FC values of 4.24 and 2.07, respectively, while PIK3C2A displayed average PSM and abundance FC values of 4.26 and 4.58. PEAK1 only met our criteria for abundance, with an average FC of 2.59 (Figure 2A,C). In the MIO-M1 PhAXA experiments, 46 kinases were reproducibly identified (n = 3). Of these, two kinases (PFKP and CDK1) met both the PSM and abundance FC criteria. PFKP displayed average PSM and abundance FC values of 2.20 and 2.10, respectively, while CDK1 displayed average PSM and abundance FC values of 5.36 and 18.72. PGK1 and STK38 only met our criteria for abundance (displaying an average abundance FC of 2.31 and 2.63, respectively), while NEK9 and TAOK1 only met our criteria for PSM (displaying an average PSM FC of 2.01 and 2.36, respectively) (Figure 2B,D). Interestingly, PIK3C2A was observed with >2 peptides in a single MIO-M1 cell PhAXA experiment, but with particularly low coverage and no observable enrichment (Supplemental File S1). Similarly, PFKP was detected in two of three R28 cell experiments, but with no observable enrichment (Supplemental File S1). CDK1, another top candidate in the MIO-M1 PhAXA experiments, was not identified in the R28 experiments (Supplemental File S1). Thus, mTOR was the only kinase amongst the identified top candidates that was reproducibly discovered in both cell lines. mTOR is a member of the PIKK kinase family with sequence similarity to PI3Ks. Thus, the results from the R28 experiments suggested that this group may target T148, at least in neurons. The top candidates from the MIO-M1 experiments were less structurally similar, suggesting possible cell-type specificity in T148 regulation or the ability of non-PIKK kinase groups/families to also target T148.

### 3.2. Bioinformatics-Based Prediction of T148-Phosphorylating Kinases

As kinases often identify substrate proteins via the recognition of a consensus sequence [65], as an additional strategy, we used bioinformatics-based tools to predict candidate kinases for T148 phosphorylation. Several programs, including iPhoPred, PhosphoSVM, PhosphoSitePlus, and NetPhos3.1, identified T148 as a phosphosite, but were unable to predict putative kinases. Kinase predictions made by HPRD PhosphoMotif Finder, Kinase Library, KinasePhos2.0, KinasePhos3.0, PhosphoNet, ScanProsite, and GPS6.0 are listed in Table 1. (see Supplemental File S2 for the full results and scores). In general, T148-phosphorylating kinase predictions were relatively weak and inconsistent between the programs. Nevertheless, there was crossover with kinase families, including those commonly involved in sHSP phosphorylation, such as AGC group kinases and p38/MAP kinases (Table 1, **bold**). Bioinformatics-based predictions for S122 more consistently pointed to AGC kinases as candidates (Supplemental File S2), which was consistent with previous reports of its phosphorylation being cAMP-dependent [66,67].

### 3.3. In Vitro Kinase Screens

We next complemented the bioinformatics and chemoproteomic approaches with in vitro kinase screens. Kinases for the preliminary screen were selected based on either being identified via bioinformatics, identified via PhAXA, known to phosphorylate sHSPs, and/or known to be expressed in the retina. Screening was performed using an HSPB4 peptide containing amino acids 142–154 (AA sequence; T148 bold: CGPKIQ**T**GLDATH). While T153 is also present in this peptide, and it cannot be fully excluded that some of the observed phosphorylation would occur at this residue, it is highly unlikely since only one previously published mass spectrometry analysis reported phosphorylation at this site and reported T148 phosphorylation as predominant [68]. We also included ATP-only controls to account for autophosphorylation, which is observed with most kinases. We compared levels of ADP generation in this control condition with those of the HSPB4 + ATP condition to determine the levels of substrate-dependent phosphorylation (i.e., ADP generation) that could be specifically attributed to kinase/substrate co-incubation and not to autophosphorylation.

**Figure 3:**
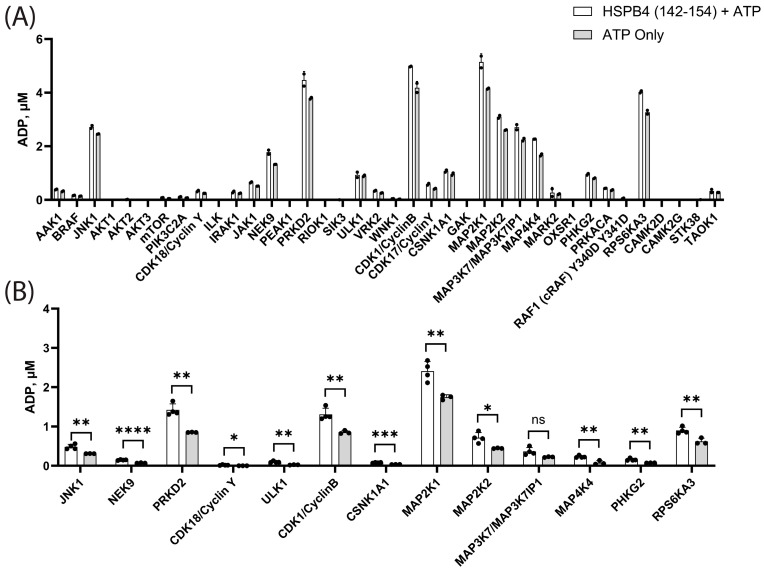
In vitro kinase screen of a T148-containing HSPB4 peptide (residues 142–154) against (**A**) 38 kinases (100 nM kinase, n = 2) or (**B**) 13 kinases that showed relatively high activity at 100 nM (10 nM kinase, n = 3 [ATP only] or n = 4 [ATP+peptide]). Data for the 10 nM screen were analyzed using an unpaired T-test using Graphpad Prism. (Error bars represent SD; ns: not significant; *: *p* < 0.05; **: *p* < 0.01; ***: *p* < 0.001; ****: *p* < 0.0001).

Under the tested conditions, 16 of the screened kinases showed weak or negligible substrate-dependent phosphorylation (Figure 3A). These, interestingly, included some of the top candidates from the PhAXA experiments, including mTOR and PIK3C2A. Of the remaining kinases examined, PRKD2, NEK9, CDK1/CyclinB, MAP2K1, MAP2K2, MAP3K7, MAP4K4, and RPS6KA3 exhibited substrate-dependent phosphorylation. A subset of kinases that showed >40% ATP–ADP conversion were tested at lower concentration to further assess their activity. PRKD2, CDK1/CyclinB, MAP2K1, and RPS6KA3 showed the highest substrate-dependent phosphorylation under these conditions (Figure 3B). Consistent with the broad range of kinases identified in the PhAXA experiments, kinases from a wide range of groups (including CAMK, CMGC, STE, TKL, AGC, and others) were able to generate T148 substrate-dependent phosphorylation in this context. Since a short HSPB4 peptide was used as the substrate in these screens, as opposed to the full-length protein during crosslinking in the PhAXA experiments, we sought to further characterize the activity of hit kinases using a full-length protein as the bait.

**Figure 4:**
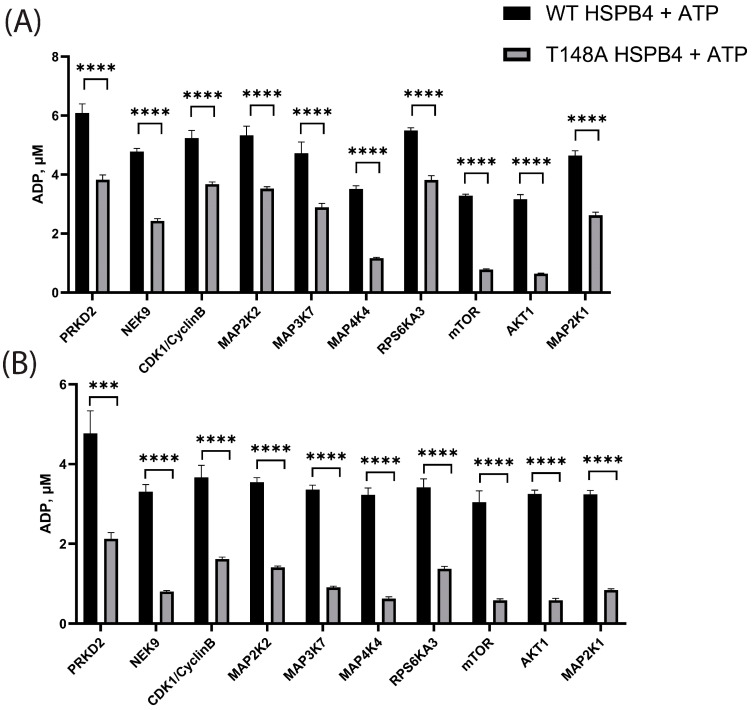
In vitro kinase screen against full-length recombinant HSPB4. (**A**) Recombinant full-length wild-type (WT) and non-phosphorylatable (T148A) HSPB4 were screened against 10 kinases (100 nM kinase, n = 4. (**B**) The screen was then repeated using a lower kinase concentration (10 nM, n = 4). (Error bars represent SD; ***: *p* < 0.001; ****: *p* < 0.0001).

For these follow-up assays, we included the top hits from the peptide screens, as well as mTOR and AKT1, and compared their phosphorylation capacity against the full-length WT protein and the T148A non-phosphorylatable HSPB4 mutant protein. T148A HSPB4 was included to account for ADP generation due to phosphorylation at other sites of HSPB4 besides T148. Of note, mTOR was included because it was the top candidate from the PhAXA experiments. Although AKT1 was not identified in the PhAXA experiments, and it too performed poorly in the peptide screen, this kinase was included due to previous studies highlighting a potential role of AKT in HSPB4 regulation [69]. Contrary to the results obtained with the peptide, mTOR and AKT1 were among the most active kinases in phosphorylating the full-length HSPB4 protein. Importantly, mTOR, AKT1, and MAP4K4 were found to exhibit high selectivity for the phosphorylation of the WT over the T148A mutant, confirming specific phosphorylation of the T148 residue (Figure 4). These findings suggest that the full-length protein, or at least portions outside of the amino acids surrounding the phosphosite, is required for T148 phosphorylation. This is consistent with the lack of strong overlap between the candidate kinase consensus sequences and the sequence surrounding T148 (Supplemental Figure S1), the poor bioinformatics prediction, and the poor performance of mTOR and PIK3C2A in the absence of the full-length HSPB4 substrate.

### 3.4. Characterization of mTOR-HSPB4 Interaction

**Figure 5:**
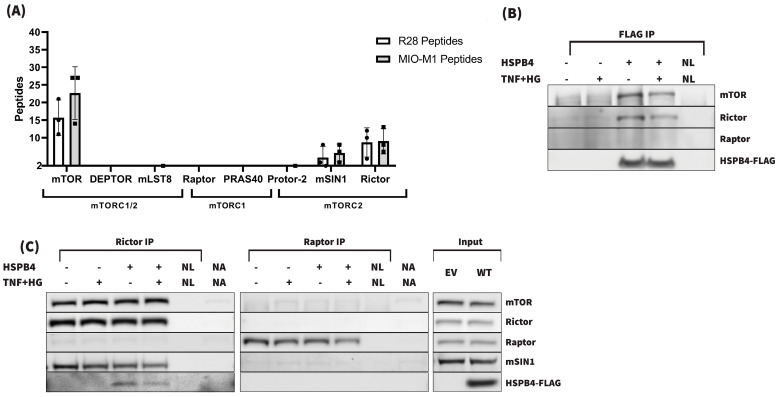
Identification and verification of mTOR–HSPB4 protein–protein interaction. (**A**) Number of peptides corresponding to mTORC1/2 component proteins identified in R28 and MIO-M1 PhAXA experiments. Error bars represent SD. (**B**) Representative FLAG IP blot validating the MS/MS results. (**C**) Representative Rictor and Raptor IP blots and corresponding input protein levels, confirming the bidirectionality of the interaction (EV—empty vector; WT—wild-type; NL—no lysate control; NA—no antibody control; TNF+HG—TNFα and high-glucose treatment).

With the results of the full-length protein screens, mTOR remained one of our top candidate kinases. mTOR functions as part of two separate complexes: mTOR complex 1 (mTORC1) and mTOR complex 2 (mTORC2). In addition to mTOR, mTORC1 is comprised of Raptor, mLST8, and PRAS40 [70]; while mTORC2 is comprised of Rictor [71,72], mSIN1/MAPKAP1 [73,74], PRR5L/Protor-2, and PRR5/Protor-1 [70,75]. These partner proteins dictate the phosphorylation and substrate interaction of mTOR [76]. Since mTOR performs its functions as part of one of these two complexes, we searched the PhAXA datasets further for mTORC1/2 complex-specific proteins. In addition to mTOR, Rictor and SIN1 were consistently identified in each R28 and MIO-M1 PhAXA experiment. Notably, neither Raptor nor any other mTORC1 complex-specific proteins were observed despite being detected in the lysate (Figure 5 and [77]). Given this seemingly highly specific mTORC2–HSPB4 interaction, we attempted to validate the MS/MS results using Western blot. Figure 5 shows the representative FLAG, Rictor, and Raptor co-IPs. The interaction was indeed verified; however, based on the transient nature of the kinase–substrate interaction, the pulldown was inconsistent and reproducibly isolating the complex was difficult without stabilizing it. To strengthen the apparently labile interaction, additional Rictor IP experiments were conducted using a shorter duration stress and in the presence of the cell-permeable general crosslinker DSP.

**Figure 6:**
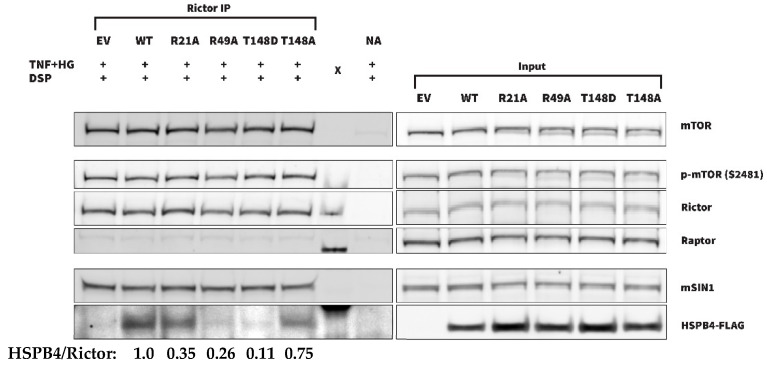
Characterization of the novel HSPB4-mTORC2 interaction by Rictor IP in the presence of a cell-permeable DSP crosslinker using several HSPB4 mutants. (EV—empty vector; WT—wild-type; NA—no antibody control; X—marker; TNF+HG—TNFα and high-glucose treatment; DSP—crosslinker treatment). For quantification, the HSPB4 IP band intensity was normalized to the HSPB4 input band, and the Rictor IP band intensity was normalized to the Rictor input band. An HSPB4/Rictor ratio was obtained by dividing these normalized values and then normalizing to the WT ratio.

Shortening the stress duration and crosslinker use (Figure 6) indeed resulted in more consistent co-IP of HSPB4 with Rictor. To further decipher the kinase–substrate vs. chaperone-driven nature of this interaction, DSP crosslinker experiments were repeated with mutant forms of HSPB4, including T148D, T148A, R21A, and R49A. R21A and R49A were included due to their respectively increased and decreased chaperone function compared with the WT HSPB4 [78]. As seen in the representative blot presented in Figure 6, WT, R21A, and T148A were found to more strongly and reproducibly associate with Rictor than the other HSPB4 mutants. The reliable identification of WT and T148A, but not T148D HSPB4 co-IP, is consistent with the interaction being strongly kinase activity-dependent. This result is indeed consistent with T148A more closely resembling the unphosphorylated protein than T148D, allowing for at least a brief interaction with Rictor that could be stabilized by the crosslinker. Of note, we also observed stronger co-IP of R21A than of R49A, both of which have a conserved T148 residue but significantly different chaperone activity, supporting that the protein–protein interaction is also at least partially chaperone function-dependent. The quantification of IP band intensity and normalization to input and Rictor levels confirms this and further suggests that the interaction is primarily kinase activity-driven. Together, our data support the kinase role of mTORC2 for T148, but also highlight that the mTORC2-HSPB4 interaction is sensitive to HSPB4 chaperone activity.

## 4. Discussion

This study is the first to characterize the kinases involved in HSPB4 T148 phosphorylation. Bioinformatics-based predictions were relatively weak but identified numerous possible candidates, including several MAP kinases and AGC group kinases (Table 1, bold). This observation was expected due to the involvement of related kinases in the phosphorylation of other sHSPs [79,80,81,82,83]. However, the PhAXA and biochemical kinase assays revealed unique kinase candidates. The PhAXA results demonstrated a consistent interaction between HSPB4 and 31 or 46 different kinases in R28 or MIO-M1 cells, respectively, with mTOR, PIK3C2A, PFKP, and CDK1 identified as the top candidates. In vitro kinase screens separately revealed T148 to be specifically phosphorylated by AKT1, CDK1, MAP2K1, MAP2K2, MAP3K7, MAP4K4, mTOR, NEK9, PRKD2, and RPS6KA3. Taken together, these results suggest that T148 is capable of being phosphorylated by numerous kinases and kinase families, albeit different from those typically associated with sHSP phosphorylation.

The identification of PIK3C2A, PFKP, and CDK1 as candidates for T148 phosphorylation in the PhAXA experiments suggests the possibility of neuron- and glia-specific regulation of T148 phosphorylation. CDK1 was only identified in MIO-M1 PhAXA experiments. PIK3C2A and PFKP, similarly, were differentially enriched in R28 and MIO-M1 cells, with each being identified in a subset of experiments in the other cell line. Even if just these experiments in which PIK3C2A (for MIO-M1) or PFKP (for R28) were identified are considered, they would still not meet the criteria [47] set for categorization as a candidate kinase. Our primary focus in this study was the retinal neuronal context, and as such, the follow-up co-IP experiments were performed in R28 cells. However, considering our previous discovery that MGCs can secrete HSPB4, which can then protect RGCs in a paracrine fashion [32], it will be valuable to further our understanding of these mechanisms and their regulation in glial cells. Additionally, the “diabetes-like” stress-dependency of the mTORC2/HSPB4 interaction is not entirely clear at this point. Our co-IP data suggest that a shorter stress duration (Figure 6) or the absence of stress (Figure 5) may in fact strengthen the interaction. Future investigations should include in-depth characterization of this interaction, including its cell- and stress-specificity, but also its relevance in vivo and modulation by other factors, such as inflammation.

Of note, while the enrichment of certain kinases in the T148C+crosslinker condition certainly points to a higher likelihood of involvement in T148 phosphorylation, it is possible that those identified with lower levels of enrichment are still *bona fide* T148-phosphorylating kinases in either or both cell lines. While highly unlikely, it is also possible that non-specific crosslinking at C131 and/or C142 of HSPB4 may have occurred during the PhAXA experiments, and further experimentation with a stepwise alanine replacement of either or both the cysteine residues would be needed to fully rule this out. However, as these residues are present in both the WT and T148C proteins, this would not explain the difference observed in the reported PhAXA analysis. Additionally, due to the relative proximity of S122 and S/T148, the slight overlap in the bioinformatics-based prediction of AGC kinases, and their seemingly similar roles in cytoprotection (at least in terms of rat astrocyte protection [30]), further studies should investigate the possibility of an overlap of S122- and S/T148-phosphorylating kinases.

AKT1 is an intriguing candidate due to its absence in the PhAXA experiments and its notable specific phosphorylation of T148 in the full-length protein (but not peptide) kinase screens. AKT has indeed been shown to phosphorylate other sHSPs and chaperone proteins, including HSPB1 [84] and HSP90 [85]. The lenticular anti-apoptotic roles of HSPB4 have also previously been attributed to interaction with the AKT pathway [69]. Conversely, our lab has shown that HSPB4-mediated and AKT-mediated neuroprotection are independent in the retinal neuronal context [31]. This could suggest that AKT is only capable of phosphorylating T148 in vitro, which would be consistent with our PhAXA findings despite expression in both cell lines used in this study. It could also reflect a redundancy in the regulation of T148 phosphorylation, with AKT only serving to phosphorylate T148 in certain contexts. In one study using full-length human HSPB4 and a microarray containing almost two-thirds of the human proteome, the only kinases to be identified as interacting with HSPB4 were AKT3, CAMK2B, RPS6KA5, and DCK [86]. Thus, it is possible for HSPB4 to interact with AKT outside of the PhAXA context, possibly in a non-kinase activity-dependent manner. Interestingly, apart from DCK, we consistently identified similar kinases (CAMK2D, CAMK2G, and RPS6KA3) in each iteration of the MIO-M1 PhAXA experiments. While they were not amongst the top candidates, this confirms their interaction with HSPB4 and again points to the possibility of cell-specific proteomes or kinase interactions.

Across the totality of our investigations, mTOR was the most promising candidate for T148 phosphorylation. In every context where the full-length HSPB4 was present, mTOR showed specific phosphorylation at the T148 residue. In full-length protein kinase screens, it was amongst the kinases showing the strongest and most specific phosphorylation at this site. In the PhAXA experiments, which also utilize full-length HSPB4 as bait and demonstrate a specific enrichment of the interaction at T148, it was the only kinase amongst all the identified top candidates (mTOR, PIK3C2A, PFKP, CDK1) that was consistently identified in both cell lines. The PhAXA and co-IP experiments strongly point to mTOR phosphorylating T148 as part of mTORC2, not mTORC1. Rictor and SIN1, two critical components of mTORC2 [71,72,73,74], but not Raptor, a critical component of mTORC1, were consistently identified in the PhAXA experiments in both cell lines. Additionally, the co-IP experiments showed an interaction between Rictor and HSPB4 but not Raptor and HSPB4. The co-IP experiments also showed an interaction between Rictor and mTOR, but little to no interaction between Raptor and mTOR. This absence of interaction could reflect an impeded interaction by the antibody but also a reduced formation of the mTORC1 complex itself in these specific cells and conditions. When the mTORC2/HSPB4 interaction was stabilized with a DSP crosslinker, we found that WT and T148A HSPB4 interacted with Rictor more strongly and consistently than T148D HSPB4, further illustrating the kinase activity-dependency of this interaction.

Unsurprisingly, the results with the R21A and R49A chaperone mutants suggest that this interaction is not solely dependent on kinase activity. The DSP crosslinker co-IP experiments showed that WT and R21A HSPB4 interacted more strongly and consistently with Rictor than R49A HSPB4. R21A and R49A HSPB4 have been shown previously to be respectively more and less effective chaperones than the WT protein [78]. This could suggest that the interaction is strengthened by the HSPB4 chaperone function and is consistent with the previous reports of chaperone-mediated mTOR regulation. Specifically, the interaction between Rictor and the substrate-recognition domain of HSP70 has been shown to underlie mTORC2 formation and activity [87]. mTORC1 formation has similarly been shown to be mediated in part by the chaperonin CCT through its interaction with mLST8 and Raptor, and its subsequent activity depends in part on these interactions [88]. mTOR also requires HSP90 and the Tel2-Tti1-Tti2 (TTT)-R2TP co-chaperone complex to fold properly [89,90]. On the other hand, mTOR has also been shown to be important in separate contexts for proper chaperone protein function, and it is possible that it similarly regulates HSPB4. For example, RSK-mediated phosphorylation of CCTβ (a subunit of the chaperonin-containing TRiC/CCT complex) is dependent on mTORC2, and upon SIN1-KO (which disrupts mTORC2 formation), CCTβ expression is reduced [91].

An interaction was still observed, albeit less frequently and consistently, between Rictor and both R49A and T148D HSPB4 during the experimental replication. R49A HSPB4, while retaining the T148 phosphosite, shows drastically reduced chaperone function [78]. T148D HSPB4, on the other hand, does not retain the T148 phosphosite and more closely mirrors the already phosphorylated isoform, but does demonstrate improved chaperone function over WT HSPB4 in some contexts [32]. The interaction between these mutants and Rictor, however infrequent, suggests that while both similarity to the unphosphorylated WT HSPB4 (T148A) and improved chaperone function (R21A) may strengthen this interaction, neither are necessary for it. Further, R21A and R49A HSPB4 have been shown to be associated with increased (for R21A) and decreased (for R49A) AKT phosphorylation on T308 and S473 [92], indicating a possible association with mTORC2 activity. Thus, it is plausible that the increased interaction seen between R21A HSPB4 and mTORC2 is indeed due to increased phosphorylation at T148 as a result of increased mTORC2 activity, as opposed to being due to improved chaperone function. Collectively, our results suggest that the HSPB4–mTORC2 interaction is driven primarily by kinase activity but is modulated and likely strengthened by HSPB4 chaperone function. A similarly multi-faceted sHSP–kinase regulatory relationship has been shown previously between HSPB1 and AKT. HSPB1 has been shown to associate with AKT, forming a complex with p38, MAPKAPK2, and AKT [93]. This interaction increases concomitantly with the level of AKT activation [94], but is also disrupted upon AKT phosphorylation of S82 on HSPB1 [84]. Thus, the AKT–HSPB1 interaction is similarly driven by both AKT kinase activity and HSPB1 chaperone function.

The identification of T148-phosphorylating kinases is a crucial next step in the development of sHSP-based therapeutics, such as for diabetic retinopathy. We have reviewed the therapeutic potential of α-crystallins more thoroughly elsewhere [95], but briefly, as T148 phosphorylation decreases substantially in diabetes and diabetic retinopathy (DR) [23], despite the upregulation of HSPB4 itself, it provides a potential target for the delay or treatment of DR, at least once the phosphorylation and protective mechanisms are further elucidated. This study provides a critical and novel investigation into the regulatory mechanisms underpinning T148 phosphorylation. We have successfully identified several kinases that are capable of phosphorylating T148 and have begun to characterize the interaction between mTORC2 and HSPB4, which appears to be largely kinase activity-driven, with the strength of the interaction increasing with increased HSPB4 chaperone function.

## Figures and Tables

**Table 1:** Bioinformatics-based program prediction results.

Phosphorylation Prediction Program	Kinase Predictions
HPRD PhosphoMotif Finder	**PKA**, **PKC**, GRK1, CK2
Kinase Library	PBK, GRK7, LKB1, skMLCK, JNK1, GAK, JNK2, JNK3, MPSK1, **p38D**, BIKE, AAK1, BRAF, MPSK1, VRK2
KinasePhos2.0	PKB, GRK, **PKC**, CDK, CK2, PDK
KinasePhos3.0	CMGC group, HT1_ARATH
PhosphoNet	MEK4, MEK7, MEK1, LKB1, SgK288, IRAK4, MEK2, IRAK1, ADCK3, MELK, IKKe, IKKb, SNRK, **PKG2**, PIM1, IKKa, **p38A**, **MAPK**
ScanProsite	CK2
GPS 6.0	TKL

## Data Availability

The original contributions presented in this study are included in the article/Supplementary Materials. Further inquiries can be directed to the corresponding author(s).

## Supplementary Materials

The following supporting information can be downloaded at: https://www.mdpi.com/article/10.3390/cells13232000/s1, Figure S1: Candidate kinase consensus sequences; Figure S2: Kinase information and ADP standard curves for kinase screens; File S1: Full PhAXA outputs; File S2: Full bioinformatics outputs.

## Abbreviations

co-IP/IP: co-immunoprecipitation/immunoprecipitation; DSP: Dithiobis (succinimidyl propionate); FC: fold-change; MIO-M1: human Müller glia cell line; mTORC1/mTORC2: mTOR complex 1/mTOR complex 2; PhAXA: phosphosite-accurate kinase-substrate crosslinking assay; R28: rat retinal neuronal cell line; rMC1: rat Müller glia cell line; sHSP: small heat shock protein; TNF-α: tumor necrosis factor alpha.
