# Supplementary material for: Novel mTORC2/HSPB4 Interaction: Role and Regulation of HSPB4 T148 Phosphorylation
Source: Cells. 2024 Dec 4;13(23):2000. doi: 10.3390/cells13232000 (PMC11640050; doi:10.3390/cells13232000)
Supplement: Supplementary file 1 [file cells-13-02000-s001.zip › Kinase Paper Supplemental Figure S1 - Candidate kinase consensus sequences.pdf]

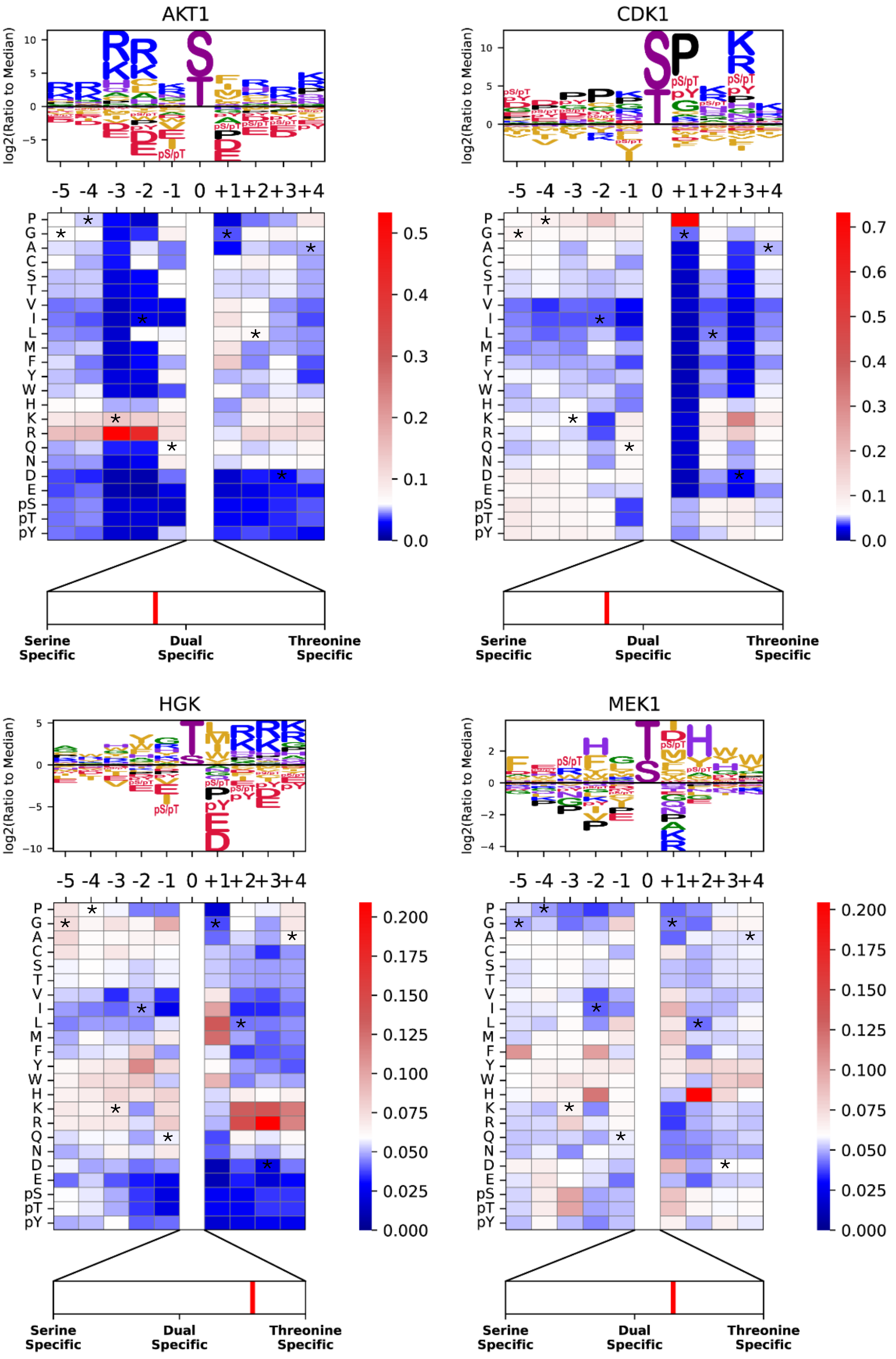
AKT1
CDK1
HGK
MEK1
log2(Ratio to Median)
-5 -4 -3 -2 -1 0 +1 +2 +3 +4
P G A C S T V I L M F Y W H K R Q N D E pS pT pY
Serine Specific
Dual Specific
Threonine Specific

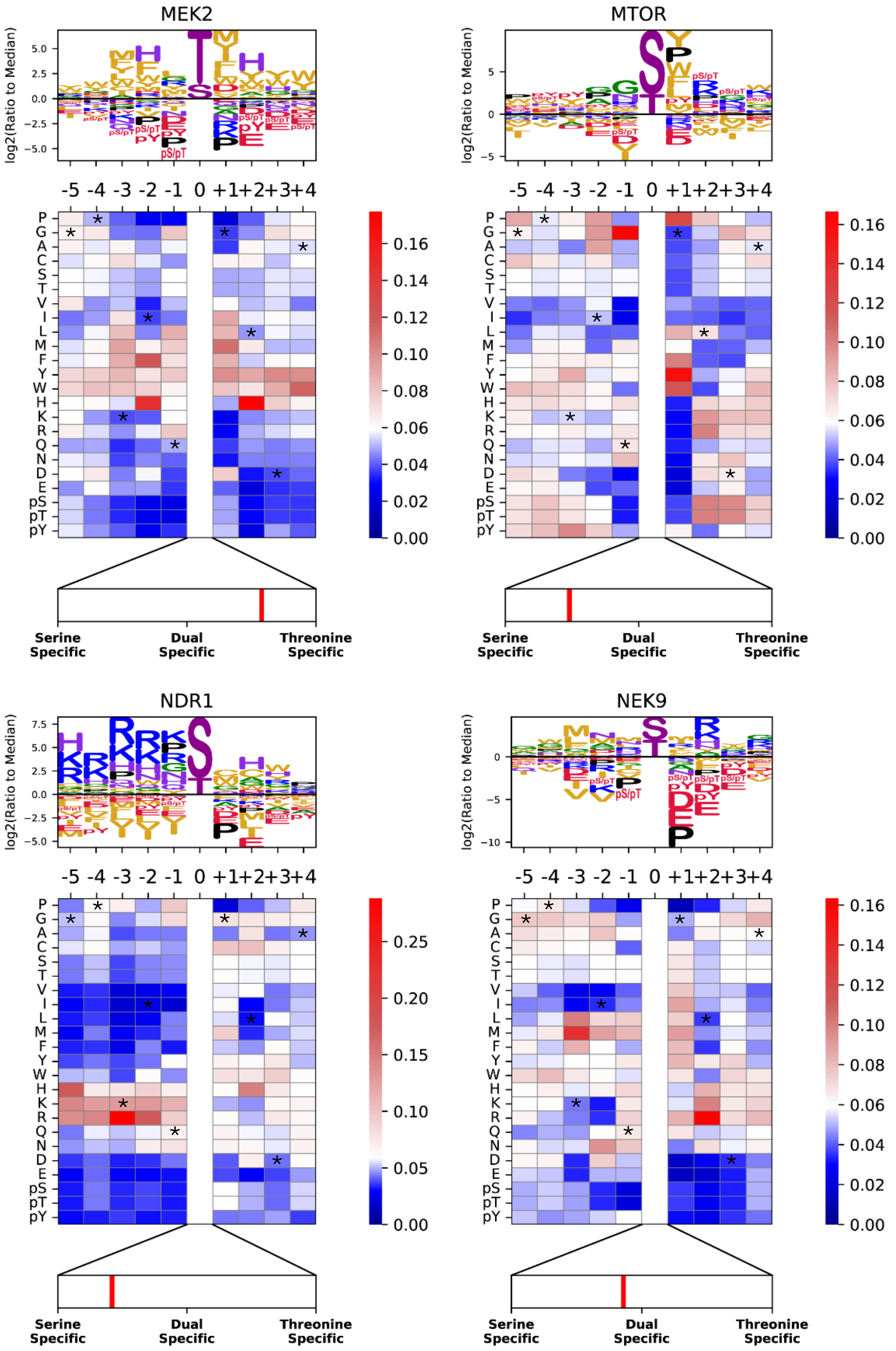

MEK2
MTOR
NDR1
NEK9
log2(Ratio to Median)
-5 -4 -3 -2 -1 0 +1 +2 +3 +4
P G A C S T V I L M F Y W H K R Q N D E pS pT pY
Serine Specific
Dual Specific
Threonine Specific

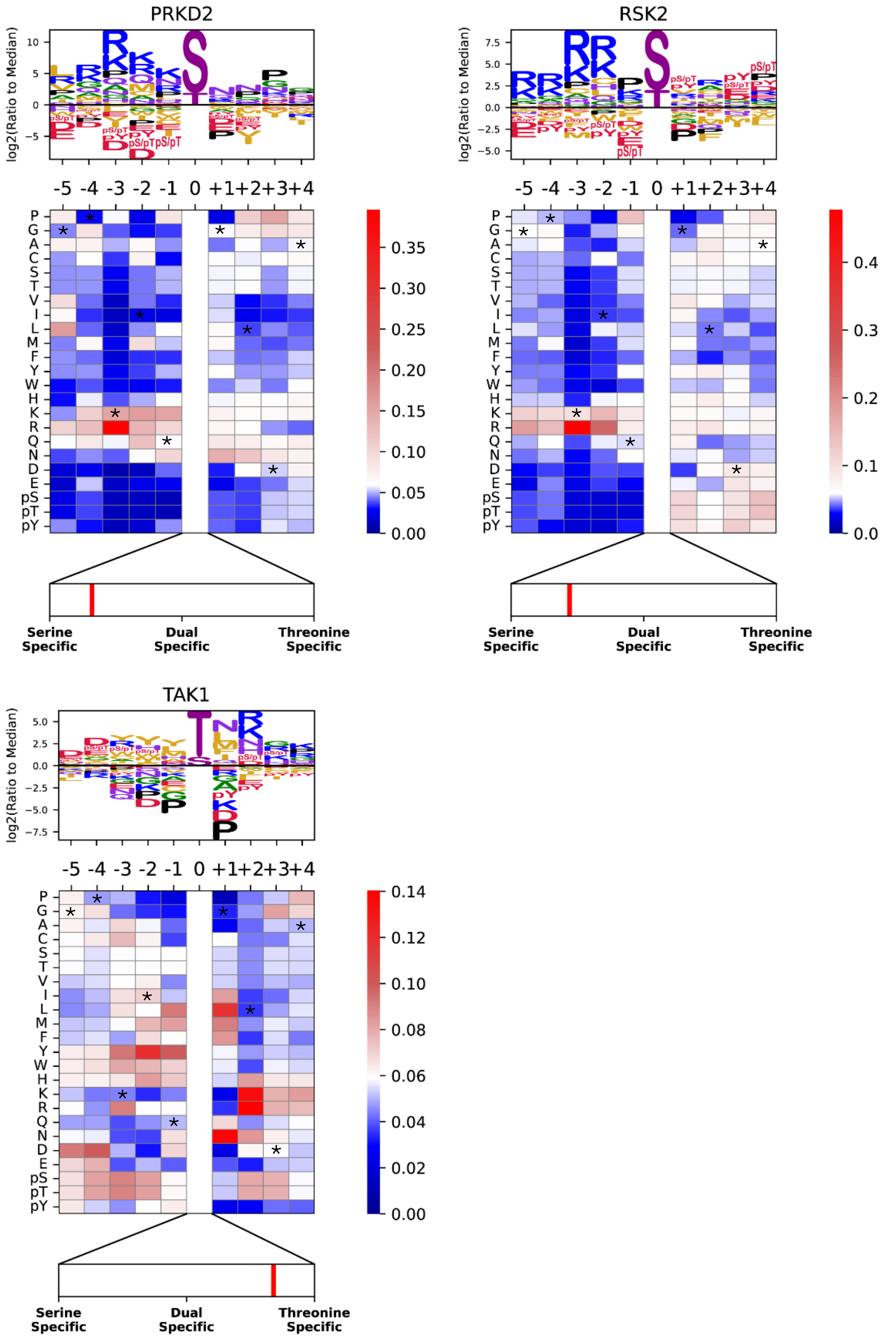

Figure S1: Candidate kinase consensus sequences obtained from https://kinase-library.phosphosite.org/site. Stars denote the amino acid sequence corresponding to that surrounding T148 in HSPB4 (GPKIQTGLDA).
