# Supplementary figures and images for: Novel mTORC2/HSPB4 Interaction: Role and Regulation of HSPB4 T148 Phosphorylation
Source: Cells. 2024 Dec 4;13(23):2000. doi: 10.3390/cells13232000 (PMC11640050; doi:10.3390/cells13232000)

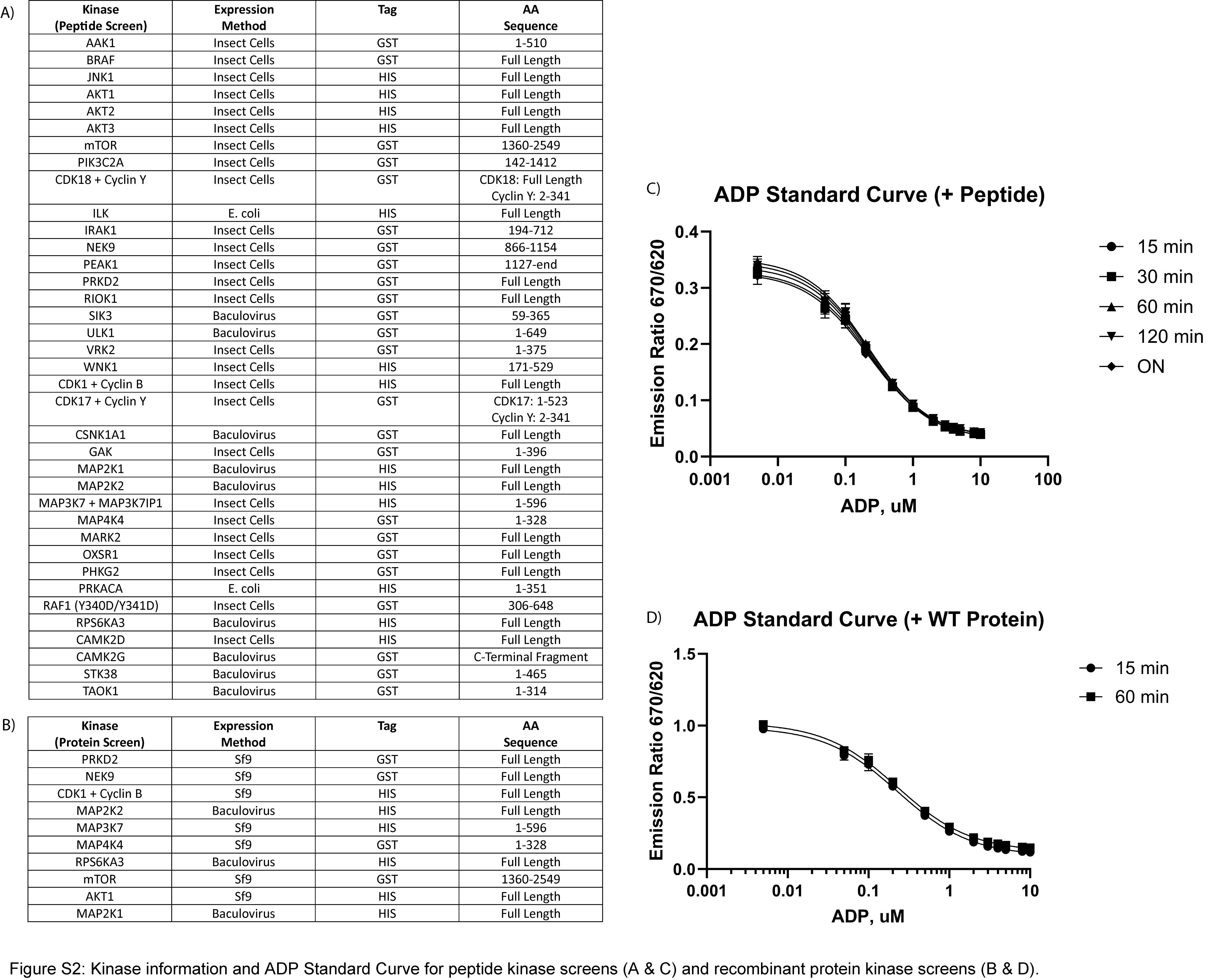

Supplement: Supplementary file 1 [file cells-13-02000-s001.zip › Kinase Paper Supplemental Figure S2 - Kinase Screen Information and Standard Curves.tif]
